# The Volunteer Functions Inventory (VFI): Adaptation and Psychometric Properties among a Portuguese Sample of Volunteers

**DOI:** 10.3390/ejihpe14040053

**Published:** 2024-03-25

**Authors:** Cátia Martins, José Tomás da Silva, Saúl Neves de Jesus, Conceição Ribeiro, Maria Dulce Estêvão, Ricardo Baptista, Cláudia Carmo, Marta Brás, Rita Santos, Cristina Nunes

**Affiliations:** 1 Psychology Research Centre (CIP), Rua de Santa Marta, 47-3º, 1169-023 Lisboa, Portugal; cgcarmo@ualg.pt (C.C.); mbras@ualg.pt (M.B.); rasantos@ualg.pt (R.S.); csnunes@ualg.pt (C.N.); 2 Faculty of Human and Social Sciences, Universidade do Algarve, Campus de Gambelas, 8005-139 Faro, Portugal; snjesus@ualg.pt (S.N.d.J.); a64413@ualg.pt (R.B.); 3 Centre for Social Studies, Faculty of Psychology and Educational Sciences, University of Coimbra, Colégio de S. Jerónimo, Largo D. Dinis, 3000-995 Coimbra, Portugal; jtsilva@fpce.uc.pt; 4 Research Centre for Tourism, Sustainability and Well-Being (CinTurs), Universidade do Algarve, Campus de Gambelas, 8005-139 Faro, Portugal; 5 Centre of Statistics and Its Applications (CEAUL), Faculty of Sciences, University of Lisboa, 1749-016 Lisboa, Portugal; cribeiro@ualg.pt; 6 Institute of Engineering, Universidade do Algarve, Campus da Penha, 8005-139 Faro, Portugal; 7 School of Health, Universidade do Algarve, Campus de Gambelas, 8005-139 Faro, Portugal; mestevao@ualg.pt

**Keywords:** motivations for volunteering, volunteering functionalist approach, psychometric properties, volunteer functions inventory

## Abstract

The Volunteer Functions Inventory (VFI) is an instrument widely used to assess volunteers’ motivation based on the Functionalist Model of Omoto and Snyder. It assesses six factors that reflect several motivational functions. The VFI has been translated into various languages and validated in different cultural contexts, but some studies have reported different factor structures (e.g., five or four factors). In the Portuguese context, previous studies have also shown inconsistent results. The aim of this study was to adapt and validate the VFI for Portuguese volunteers, testing several alternative models (nine models) using confirmatory factor analysis. The sample comprised 468 volunteers (76.3% women), aged from 13 to 81 years (*M* = 36.66, *SD* = 14.93). The results support the original interrelated six-factor model as the best-fitting one. The VFI showed good internal consistency and convergent validity. Significant correlations were found between the VFI factors, organizational commitment, and volunteers’ satisfaction. Overall, the six-factor VFI is a valid and reliable tool for measuring the motivational functions of Portuguese volunteers, with implications for practice and research in the volunteering field.

## 1. Introduction

According to a United Nations report [1], there are over 862 million volunteers around the world, who represent a fundamental human work force [2]. Volunteering has been defined as a non-mandatory prosocial behavior [3,4], planned and sustained over time, with no expectation of monetary reward [5,6,7], that benefits strangers [4] and occurs within an organizational setting [4,8,9].

Volunteering is a very attractive phenomenon for organizations and researchers from several areas [10], and is also relevant for local and national government policymaking [11]. Therefore, an important question is to understand why people dedicate part of their time to activities without any type of monetary compensation [12,13], which poses a challenge for non-profit organizations [5,14]. Considering the features of the current daily life, and the role that volunteering can represent, e.g., [15], it remains difficult to recruit volunteers and to retain them in a more steady and sustainable collaboration, as opposed to episodic contributions [11].

Volunteer motivation is one of the most common factors that researchers have examined to understand the reasons why people volunteer (e.g., altruism, social support, organizational integration, and engagement) [16]. Based on this factor, Clary, Snyder, and Omoto [3,5,17] proposed the Volunteer Functionalist Theory or Volunteer Process Model (VPM) [13], a conceptual model that views volunteering as a voluntary, long-term, and dynamic form of helping that involves three stages: antecedents, experiences, and consequences [3,13]. According to this model, the motivation to start and sustain volunteer work is a process that depends on the psychological functions that individuals seek to fulfil through their activities, such that different activities can serve different functions, and vice versa [18,19]. Thus, Clary and collaborators [5,8] suggested that people choose to volunteer because of the internal needs or functions that volunteering satisfies, which they call the functional motives. The model also implies that these motives are significant predictors of whether volunteers intend to continue or quit their activities [16].

Considering this model [20], a self-report instrument is used to measure the extent to which volunteers’ current organizations satisfy each of the six motives identified (i.e., values, understanding, social, career, protective, and enhancement), named the Volunteer Functions Inventory (VFI) [8]. Although there are other instruments available to assess volunteers’ motivations (e.g., attitudes toward helping others [21]; the Helping Attitudes Scale, [22]; the Motivation to Volunteer Scale [23]; and the Volunteer Motivation Scale [14,24]), the VFI is the most widely used one [16,20,25].

The original instrument was designed to measure five functions of volunteering (values, understanding, personal development, community concern, and esteem enhancement) with 25 items [3]. A revised version added a sixth function (protective) and increased the number of items to 30 [5,8]. This six-function survey is the most widely used measure of volunteer motivation [20]. The functions are defined as follows: *values* reflects the expression or enactment of one’s core values (e.g., humanitarian, altruistic); *understanding* refers to the desire to learn more and to enhance one’s skills and experiences; *enhancement* represents a self-oriented motivation, where the volunteer seeks to achieve psychological growth and development (e.g., self-knowledge, self-esteem), and to experience positive emotions; *career* is also a self-oriented and instrumental motivation, where the volunteer aims to gain career-related benefits, such as professional and academic knowledge and experience; *social* indicates that an individual volunteers to increase and improve their social relationships and interactions; and *protective*, an ego-defensive motivation, denotes the intention to reduce negative feelings (e.g., guilt, frustration) and/or to escape from personal problems [5,16].

Chacón et al. [16] conducted a systematic review of 26 studies that performed an internal structure analysis (i.e., factor analysis) of the Volunteer Functions Inventory (VFI). They found that 17 studies supported the six-factor model proposed by the original authors of the VFI, e.g., [12,26], while the remaining reported different factor structures. Some studies reported five factors, e.g., [25,27], four factors, e.g., [28,29] (some eliminated items from the original scale, e.g., [29,30]), and seven factors [31]. A more recent study [32] also identified a five-factor model, merging the enhancement and protective subscales of the VFI. Despite the variability in the factor structures, the original authors of the VFI [8,20] and other researchers [20,26] have argued that the six-factor model is still a valid and reliable measure of volunteers’ motivation. To test this claim, Wu et al. [26] compared six models (i.e., first-order, general one-factor, first-order two-uncorrelated factor, first-order two-correlated factor, first-order six-uncorrelated factor, first-order six-correlated factor, and second-order one-general factor loaded on six first-order factors) and confirmed that the first-order, six-correlated-factor model had the best fit to the data. Based on this information, we proposed that:

**H_1_:**  *The intercorrelated six-factor model will have the best fit.*

**H_2_:**  *VFI will reveal good internal consistency and discriminant validity.*

Among the six factors of the VFI, some are more relevant than others. Chacón et al. [16] reported in their systematic review that values (*M* = 5.21; *SD* = 1.35), and understanding (*M* = 4.26; *SD* = 1.49) had the highest scores, while career (*M* = 2.89; *SD* = 1.23) and protective (*M* = 2.82; *SD* = 1.46) had the lowest scores, indicating that other-oriented factors (e.g., values) were more prevalent than self-oriented factors (e.g., career). They also noted that values had the lowest reliability (α = 0.78), while the other subscales had good internal validity, ranging from 0.82 to 0.84. Based on these, we proposed that:

**H_3_:**  *Volunteers will reveal higher mean scores in the understanding and values functions and lower mean scores in the career and protective functions.*

The literature on the Volunteer Functions Inventory (VFI) research has examined different age groups, such as older adults, e.g., [33,34], adolescents and young adults, e.g., [35], and mixed-age samples, e.g., [36]. Chacón et al. [16] reported that volunteers under 40 years old scored higher on the career and understanding subscales than older volunteers, and this difference was statistically significant. This is in line with a meta-analysis [20] that found that student samples showed stronger associations between the career subscale and satisfaction and commitment with volunteering. For Gen Z volunteers, values and career were the most important factors [27,36,37]. For older-age samples, the enhancement subscale had stronger effects, while the social, understanding, and values subscales had weaker effects [20]. Based on this information, we proposed that:

**H_4_:**  *Younger volunteers will reveal higher scores of the career, values, and understanding sub-scales.*

**H_5_:**  *Older volunteers will reveal higher scores of the enhancement sub-scale.*

Okun et al. [38] reported that when considering gender differences in motives for volunteering, women tended to score higher on the VFI than men, which is supported by Pearl and Christensen [35] who referred that, apart from the social function, women had higher values in all the other functions. Fletcher and Major [39] pointed out that both men and women showed similar patterns of the relative importance of the six VFI functions. Chacón et al. [16] split the studies with respect to volunteers’ gender and found that women had higher scores in the social subscale. Zhou and Muscente [20] also identified a moderating effect of gender in four of the six functions (e.g., males tended to grade higher on the career, protective, enhancement, and social subscales). It should be noted that the differences between gender (and also culture/race) in VFI have been little researched [35], and contradictory data are still being found, which proves the need to strengthen research in this field. Based on this information, we proposed that:

**H_6_:**  *Feminine volunteers will have higher scores in the social sub-scale.*

**H_7_:**  *Male volunteers will have higher scores in the career, protective, and enhancement sub-scales.*

The functionalist framework [3,5,13,17] proposes that volunteer motivation is related to various outcomes, such as satisfaction, engagement, and commitment. Previous studies have supported this hypothesis by finding several associations between these variables, e.g., [20,40]. Volunteering is a non-remunerative activity that requires involvement with organizations and tasks [5,6,7]. Therefore, it is important that volunteers experience satisfaction and engagement in their roles, which may depend on the alignment of their tasks and motivations, e.g., [3,24,40].

**H_8_:**  *VFI will be positively associated with organizational commitment, engagement, and volunteers’ satisfaction.*

The Volunteer Functions Inventory (VFI) is an instrument widely used to measure the motives of volunteers across different contexts and cultures [16]. It has been translated into various languages, such as Brazilian [18], Chinese [26], Dutch [12], German [41], Polish [42], Portuguese [29], Serbian [43], and Spanish [44]. However, in the Portuguese context, there is a scarcity of research using the VFI. Souza et al. [45] applied the VFI to a small sample of 51 female volunteers from Portugal and Brazil who volunteered in cancer services, but they did not conduct any rigorous statistical analysis or draw any conclusions about the instrument’s structure. Ferreira et al. [29] developed a study to examine the configuration of volunteer motivation using the VFI. They translated and adapted the instrument to the Portuguese language and performed an exploratory factor analysis that yielded four factors, after excluding five items with low factor loadings (items 5, 11, 16, 17, and 22, from four original subscales). They labelled the factors as development and learning (9 items; α = 0.90), belonging and protection (9 items; α = 0.87), career recognition (5 items; α = 0.85), and altruism (2 items; α = 0.60). They also found significant associations between the factors and demographic variables, such as the age and education level (a negative correlation with belonging and protection). Monteiro et al. [46] assessed the motives of 53 volunteers, using an adaptation of the VFI for the Portuguese population, which was developed in an unpublished academic study [47].

Considering the relevance of VFI for measuring the motivations of volunteers, the aim of this study is to adapt and validate the VFI for a Portuguese sample of volunteers. Based on previous studies [16,20,26,32,34], we compared nine models of the VFI (i.e., one-factor, two-interrelated factor, two-independent factor, four-factor, five-factor, six-intercorrelated factor original structure, six-intercorrelated factor with item 29 assigned to the social dimension, six-independent factor, and six-independent factor first-order and second-order), reflecting the different structures reported in the literature. We also evaluated the internal consistency, sensitivity, and convergent validity of the VFI in relation to volunteer satisfaction, commitment, and engagement.

## 2. Materials and Methods

### 2.1. Sample

We recruited 468 volunteers (76.3% women, age range = 13–81 years, M = 36.66, SD = 14.93) with various educational backgrounds: 0.21% had no formal education, 12.42% had completed primary school, 32.76% had finished high school, and 54.18% had obtained a university degree. The volunteers had been working for an average of 44.05 months (SD = 65.75, range = 1–600 months), mostly on a weekly basis (43.6%), in the following areas: social (87.6%), health (1.4%), cultural (1.4%), and administrative (9.6%).

### 2.2. Instruments

The Volunteer Functions Inventory (VFI) [3,8] was translated and adapted in the present study. It consists of 30 items that assess six functions: values (e.g., “I can do something for a cause that is important to me”), understanding (e.g., “Volunteering allows me to gain a new perspective on things.”), social (e.g., “My friends volunteer”), career (e.g., “Volunteering can help me to get my foot in the door at a place where I would like to work”), protective (e.g., “Doing volunteer work relieves me of some of the guilt over being more fortunate than others”), and enhancement (e.g., “Volunteering increases my self-esteem”). Each item was rated on a seven-point scale ranging from 1 (not at all important/accurate) to 7 (extremely important/accurate). We calculated the scores for each function by averaging the corresponding items, with higher scores indicating a higher importance of that function.

To evaluate the satisfaction of volunteers, we administered the Portuguese version validated by Martins et al. [24] of the Volunteer Satisfaction Survey (VSS) developed by Vecina et al. [48]. The VSS is a 17-item instrument that measures 3 dimensions of satisfaction: satisfaction of motivations (six items;, e.g., “The tasks I usually perform as a volunteer allow me to establish social relationships with different people”; α = 0.82), satisfaction with the management of the organization (seven items;, e.g., “Satisfaction with the overall management of the organization”; α = 0.92), and satisfaction with the tasks (four items;, e.g., “I am satisfied with the effectiveness with which I perform the tasks assigned to me”; α = 0.81). Participants responded to each item using a seven-point Likert-type scale from 1 (very dissatisfied) to 7 (very satisfied). Higher scores indicated a greater satisfaction with each dimension.

To measure the volunteers’ engagement, we used the Utrecht Work Engagement Scale (UWES-9, Schaufeli et al. [49]), a nine-item instrument that evaluates three dimensions: vigor (e.g., “At my job, I feel strong and vigorous”; α = 0.87), dedication (e.g., “My job inspires me”; α = 0.85), and absorption (e.g., “I am immersed in my work”; α = 0.82). The responses were given on a seven-point Likert-type scale from 0 (never) to 6 (every day), with higher scores indicating higher levels of work engagement.

We also used the Organizational Commitment Questionnaire (OCQ, Mowday et al. [50]; Portuguese version by Carochinho et al. [51]) to assess the volunteers’ commitment and attachment to the organization. The OCQ consists of 15 items that measure 3 dimensions: affective (e.g., “I am willing to put in a great deal of effort beyond that normally expected in order to help this company to be successful”; α = 0.85), cognitive (e.g., “I feel little loyalty to this organization”; α = 0.65), and behavioral (e.g., I really care about the fate of this company”; α = −0.21). A seven-point Likert-type scale (1 = strongly disagree; 7 = strongly agree) was used, with higher scores indicating higher levels of organizational commitment.

Additionally, a sociodemographic questionnaire was also used to collect information on gender, age, and volunteering-related variables (e.g., organization, area, volunteering dedication).

### 2.3. Procedures

The Psychology and Sciences Education Department of the University of Algarve approved this study (N.º 59/14.02.2007).

We translated and back-translated the VFI from English to Portuguese, and then the final version was reviewed by an experience Portuguese teacher [52]. Then, we recruited volunteers using snowball sampling. We informed the participants about the study objectives, the voluntary and confidential nature of their participation, and their right to withdraw at any time.

### 2.4. Data Analysis

We used IBM SPSS 28.0.1.0 (IBM Corp., Chicago, IL, USA), R version 4.2.1, and the *lavaan* package (v0.6-12) [53,54,55] to process the data and compute descriptive statistics (e.g., mean, standard deviation, kurtosis, skewness). We considered absolute skewness values above 3 and kurtosis values above 7 as severe violations of normality [56]. We performed a Confirmatory Factor Analysis (CFA) to test the factorial structure of the Portuguese version of the VFI. Since the data were not normally distributed, we used the MVN package of R to check the univariate and multivariate normality and the DWLS (diagonally weighted least squares) estimation method in the *lavaan* (latent variable analysis) package of R to account for the non-normality of the items. We obtained the following goodness of fit indices: the chi-square statistic divided by the degrees of freedom, the comparative fit index (CFI), the goodness of fit index (GFI), the incremental fit index (IFI), the Tucker–Lewis fit index (TLI), the root mean square error of approximation (RMSEA), and the standardized root mean square residual (SRMR). We considered the model to have a very good fit if the chi-square statistic divided by the degrees of freedom was below 5, the fit indexes were above 0.90, the RMSEA was below or equal to 0.05, and the SRMR was below or equal to 0.08. We calculated the Cronbach alpha to assess the internal consistency and considered scores between 0.60 and 0.70 as satisfactory, above 0.70 as adequate, and above 0.90 as excellent. We eliminated the subscale if the score was below 0.50. We also obtained composite reliability (CR) and considered values above 0.7 as indicators of good reliability [57]. For convergent validity, we considered the following indicators: CR values above 0.7, standardized factor loadings above 0.4, and average variance extracted (AVE) above 0.5 [57,58]. For discriminant validity, we considered the following indicators: convergent validity being established, a correlation between two constructs below 0.9, and the square root of the AVE being greater than the correlation between two factors (also known as the Fornell–Larcker criterion) [57,58,59]. We used Pearson correlations to analyze the convergent validity and considered scores between 0.20 and 0.40 as low, between 0.40 and 0.60 as moderate, and between 0.60 and 0.80 as high. We considered the *p*-value to be significant if *p* < 0.05 [60].

## 3. Results

### 3.1. Descriptive Analysis

The mean values of the VFI items (Table 1) varied from 2.39 (item 11, *SD* = 1.80) to 6.48 (item 19, *SD* = 0.96), indicating heterogeneity in the responses. The response scale ranged from 1 to 7, and all items had the full range of possible values. The univariate skewness and kurtosis values (−2.41 to 1.18 and −1.42 to 6.67, respectively) did not suggest a serious departure from normality. However, the multivariate skewness and kurtosis values (163.33 and 1137.70, respectively) indicated a non-normality of the data. All univariate and multivariate tests in the MVN package of R confirmed this finding. Therefore, we used the DWLS estimation method of the *lavaan* R package.

### 3.2. Internal Structure Analysis

As shown in Table 2, the six-factor model with five items per factor had the best-fit indices. The model had a chi-square ratio of 2.30, RMSEA = 0.05, CFI = 0.98, TLI = 0.97, and SRMR = 0.07, which all met the criteria for a good fit.

As expected, all estimated factor loadings exceeded the recommended cutoff point (<0.50), revealing proper individual reliability values (*R*^2^ ≥ 0.25), supporting the factorial validity (Table 3).

### 3.3. VFI Internal Consistency

As presented in Table 4, all six constructs had satisfactory CR values ranging from 0.70 to 0.90, despite the lowest alpha value being 0.69. The MIIC values indicated that the items were sufficiently correlated, although some values exceeded 0.5 (social = 0.53, career = 0.66, and enhancement = 0.52). The CITCR values met the minimum threshold of 0.3, confirming the acceptable internal consistency of the constructs.

### 3.4. VFI Convergent Validity

The convergent validity of the measures was evaluated by examining the CR, the standardized factor loadings, and the AVE (Table 5). The standardized factor loadings of the 30 items on the six factors were all above 0.40, ranging from 0.41 to 0.90. The AVE values varied from 0.36 to 0.66, with two values below the cut-off of 0.50 for factor one (0.36) and factor six (0.43). However, these values were acceptable, as the CR values of the corresponding factors were higher than 0.6 [58]. The CR values of the six constructs ranged from 0.70 to 0.90, exceeding the recommended level. The AVE values of the other factors were equal to or higher than 0.50. Therefore, all measures showed convergent validity.

Discriminant validity was assessed by comparing the factor correlations with the square root of the average variance extracted (AVE) for each factor. The factor correlations ranged from 0.27 to 0.82, which were lower than the recommended threshold of 0.9, indicating adequate discriminant validity. However, some factors had a square root of AVE that was lower than the correlation with other factors. Specifically, the square root of AVE for factor one was 0.60, while its correlation with factor two was 0.81 and with factor six was 0.68. Similarly, the square root of AVE for factor two was 0.70, while its correlation with factor six was 0.77. The square root of AVE for factor six was 0.67, while its correlation with factor one was 0.68, with factor two was 0.77, and with factor five was 0.67. Following the suggestion of Voorhees et al. [61] (p. 124), supported by Rönkkö and Cho [59], this criterion is “a very conservative test”, and therefore we also examined the confidence intervals for the factor correlations. They proposed that if the 95% upper limit (UL) of the factor correlation is equal to or greater than 1, there is a severe problem of discriminant validity; if 0.90 ≤ UL < 1, there is a moderate problem; if 0.80 ≤ UL < 0.90, there is a marginal problem; and if UL < 0.80, there is no problem. Based on this criterion, the results in Table 6 show that there was no severe or moderate problem of discriminant validity in this study, although some factors had a marginal problem.

As shown in Table 6, the upper limit (UL) of the confidence interval for the correlation between factor 1 and factor 2 was 0.81, suggesting a potential issue of discriminant validity (moderate problem). However, all other UL values were below 0.80, indicating that the six factors were sufficiently distinct from each other. Therefore, we can conclude that the discriminant validity criterion was met for the six-factor model.

**Figure 1:**
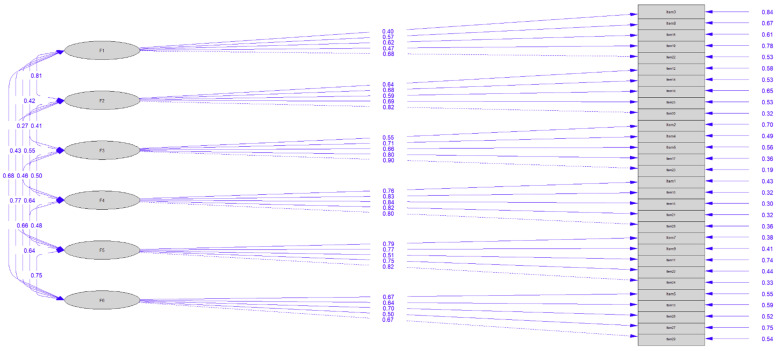
VFI CFA model results.

The confirmatory factor analysis (CFA) yielded a six-factor solution for the VFI, as shown in Figure 1. The factors were labelled as values, understanding, social, career, protective, and enhancement.

The correlations between the VFI (i.e., motivation) and other relevant constructs (Table 7) were computed. The values and understanding factors revealed positive and significant correlations with all dimensions of the organizational commitment (affective: *r_values_* = 0.37, *p* < 0.01; *r_understanding_* = 0.50, *p* < 0.01; cognitive; *r_values_* = 0.23, *p* < 0.01; *r_understanding_* = 0.16, *p* < 0.05; compromise: *r_values_* = 0.36, *p* < 0.01; *r_understanding_* = 0.42, *p* < 0.01), with the psychological engagement (UWES: *r_values_* = 0.33, *p* < 0.01; *r_understanding_* = 0.37, *p* < 0.01), and volunteer’s satisfaction (VSS: *r_values_* = 0.32, *p* < 0.01; *r_understanding_* = 0.47, *p* < 0.01). The enhancement factor presented positive correlations with most of the dimensions of the organizational commitment (affective: *r* = 0.52, *p* < 0.01; compromise: *r* = 0.41, *p* < 0.01), psychological engagement (UWES: *r* = 0.28, *p* < 0.01), and volunteer’s satisfaction (VSS: *r* = 0.45, *p* < 0.01). The career and protective factors showed positive and significant correlations the affective dimension of the organizational commitment (*r_career_* = 0.23, *p* < 0.01; *r_protective_* = 0.22, *p* < 0.01) and with psychological engagement (UWES: *r_career_* = 0.15, *p* < 0.05; *r_protective_* = 0.19, *p* < 0.05) and volunteer’s satisfaction (VSS: *r_career_* = 0.32, *p* < 0.01; *r_protective_* = 0.27, *p* < 0.01). The correlations’ magnitude varied between small and moderate (*r* = 0.15–0.52), revealing relations between the constructs, but also an independency.

Regarding age, the results show small negative and significant correlations in the sub-scales understanding (*r* = −0.18, *p* < 0.01), protective (*r* = −0.09, *p* < 0.05), and enhancement functions (*r* = −0.15, *p* < 0.01), and a moderate correlation in the career subscale (*r* = −0.50, *p* < 0.01), revealing that the younger volunteers reported higher scores.

Considering the scores of the VFI sub-scales (Table 8), the results reveal higher mean scores in values (*M* = 5.86, *SD* = 0.91, *Range* = 1.40–7.00), understanding (*M* = 5.82, *SD* = 1.02, *Range* = 2.00–7.00), and enhancement (*M* = 5.10, *SD* = 1.27, *Range* = 1.00–7.00). The others subscales showed proximal mean scores (*M_Career_* = 3.84, *SD_Career_* = 0.81, *Range_Career_* = 1.00–7.00; *M_Social_* = 3.73, *SD_Social_* = 1.52, *Range_Social_* = 1.00–7.00; *M_Protective_* = 3.64, *SD_Protective_* = 1.57, *Range_Protective_* = 1.00–7.00).

With respect to the gender comparison (Table 8), only the social (*t* = 2.68, *p* = 0.004, *d* = 0.29) and enhancement subscales (*t* = 1.74, *p* = 0.042, *d* = 0.19) revealed significant differences (*p* < 0.05), and in both cases the male volunteers showed higher mean scores than the female volunteers (social: *M*_Female_ = 3.62, *SD*_Female_ = 1.48; *M*_Male_ = 4.06, *SD*_Male_ = 1.60; enhancement: *M*_Female_ = 5.04, *SD*_Female_ = 1.26; *M*_Male_ = 5.28, *SD*_Male_ = 1.27). Understanding (*t* = −1.72, *p* = 0.079, *d* = −0.15) and protective functions (*t* = 1.52, *p* = 0.065, *d* = 0.17) were marginally significant (*p* < 0.01), and female volunteers only revealed higher scores in the understanding function (*M*_Female_ = 5.86, *SD*_Female_ = 1.03; *M*_Male_ = 5.70, *SD*_Male_ = 0.98; protective: *M*_Female_ = 3.57, *SD*_Female_ = 1.57; *M*_Male_ = 3.83, *SD*_Male_ = 1.57).

## 4. Discussion

Volunteering is a significant phenomenon for current and future societies, as it affects individuals, organizations, and communities [10,11]. As a form of prosocial behavior [3,4] that is intentional, long-term, unpaid [5,6,7], and directed towards strangers [4], it is crucial for organizations, professionals, and researchers to evaluate and monitor volunteers’ motivations so as to enhance their fit and engagement with the activities.

To this end, several instruments can assist in this evaluation (e.g., attitudes toward helping others [21]; the Helping Attitudes Scale [22]; the Motivation to Volunteer Scale [23]; and the Volunteer Motivation Scale [14,24]), but the Volunteers Function Inventory is one of the most relevant and widely used [16,20,25]. Considering that it is easier to cross-culturally adapt than create a new instrument, we translated and back-translated the VFI from English to Portuguese and its final version was reviewed by a Portuguese specialist to ensure that the new version was properly adapted [62].

Several studies have examined the VFI’s internal structure (see [16,26]), revealing different final models from the original one—a six-intercorrelated factor [5,8]. In the Portuguese context, there have been some attempts to adapt and test the VFI [29,45], but they either lacked rigorous statistical analysis [45] or reported a different structure from the original (i.e., a four-factor model) [29], indicating the need for further research. Thus, this study aimed to adapt and validate the VFI in a sample of Portuguese volunteers. Following previous research [16,20,26,32,34], we intended to test nine models (i.e., one-factor, two-interrelated factor, two-independent factor, four-factor, five-factor, six-intercorrelated factor original structure, six-intercorrelated factor with item 29 assigned to the social dimension, six-independent factor and six-independent factor first-order and second-order), as well as to assess the VFI’s internal reliability, sensibility, and convergent validity (regarding volunteer satisfaction, commitment, and engagement).

We performed an exploratory analysis to evaluate the data and checked univariate and multivariate normality. We found that the data deviated from normality and used the DWLS estimation method of the *lavaan* R package to account for the non-normality of the items. Several models from the literature were tested (i.e., nine models), and we found that the six-factor model had the best fit. This confirms H_1_, as our data support the original six-factor structure proposed by Clary et al. [5,8].

The results show acceptable internal consistency, convergent validity, and discriminant validity for the six-intercorrelated factor model. This confirms H_2_, indicating that the VFI is a valid and reliable measure of volunteers’ functional motivations in this context and tasks. These results are consistent with those of Chacón et al. [16], who reported in their systematic review that most factor analyses of the VFI, e.g., [12,26], retained the six factors of the original structure. This suggests that the VFI has high dimensional stability. Regarding internal consistency, all factors had adequate scores, with values and enhancement being the lowest.

The functions with the highest scores were values, understanding, and enhancement, while the rest had moderate scores. These results partly support H_3_, which may depend on the sample characteristics (e.g., age, education level). However, they also confirm the consistent finding that volunteers are motivated by their values, learning, and personal growth [3,5,16,63]. In a study developed by Vecina and Manzana [63], the authors cross-referenced results from the VFI with open-ended questions and found that despite the type of assessment, the more identified motivations were values, understanding, and enhancement. However, the study also suggested that the others function did not receive the same level of confirmation, whereby some structural changes were suggested (e.g., the removal of the social and protective dimensions, and the insertion of new categories).

Age was negatively associated with the understanding, protective, enhancement, and career functions, indicating that younger volunteers scored higher on these functions (H_4_ is supported but not H_5_). This is in line with the VFI systematic reviews [16,20], which suggested that younger volunteers are more influenced by the volunteering contexts and opportunities for personal and social development. The results of this psychometric study validate the VFI for young adults and adults.

Gender differences were significant for the social and enhancement functions, but opposite from the hypotheses formulated (H_6_ was not supported while H_7_ was partially supported), with male volunteers scoring higher on both functions. These results are closer to those of Zhou and Muscente’s meta-analysis [20], which found a gender moderating effect on four of the six functions (i.e., career, protective, enhancement, and social).

Consistent with our expectations, the results indicate positive and significant correlations between all VFI sub-scales and the affective dimension of organizational commitment, as well as volunteers’ satisfaction and psychological engagement. Additionally, some sub-scales show positive and significant correlations with the cognitive (values and understanding) and the compromise (values, understanding and enhancement) dimensions of organizational commitment. Thus, H_8_ was supported by our data. This finding highlights the importance of the processes proposed by the functionalist framework [3,5,13,17], and also suggests that volunteers’ functional motivations are relevant for both professionals and organizations, as they can affect their engagement, commitment, satisfaction, and retention [9,33].

Several studies have highlighted the importance of understanding the volunteers’ features before they start volunteering. Evidence indicates that their personal interests (e.g., [64,65]), motives for volunteering [64,65,66], gender roles [64], and ages [64,65] have a strong influence on their involvement in and satisfaction with volunteering. In this sense, research into the nature of motivation for volunteering (e.g., [38,67]) highlights the positive relationship between the identification of motives for volunteering, through the use of the VFI, and the recruitment process, satisfaction, commitment, and motivation. Thus, we consider that this Portuguese version of the VFI can be a useful tool for professionals to assess volunteers’ motivations and to consider their profile in the strategic management of the host organization. Satisfied volunteers have the potential to engage in a richer and more productive way, as well as to sustain longer-term volunteering, thus contributing to their own personal development and to the transformation of organizations and communities.

However, some limitations of this study should be acknowledged. First, our sample exhibited some heterogeneity (e.g., age, duration of volunteering) that might have influenced some of the results obtained. Therefore, future studies should examine these factors and their relation to volunteers’ motivation in more detail. Another relevant aspect refers to the use of the VFI, without cross-referencing the volunteers’ motivation with other colleting techniques (e.g., narratives, open-ended questions), which could affect the actualization of this measure [63]. Therefore, future research should consider mixed methods and also the devolution of the results to the participants, as a confirmatory or validation strategy.

Despite these limitations, the present research demonstrated the validity and reliability of the Portuguese version of the VFI. Our conclusions imply that it could be a valuable tool for researchers and professionals interested in motivation for volunteering in the Portuguese context.

## Figures and Tables

**Table 1:** Descriptive statistics of the VFI items.

VFI Item	*M*	*SD*	*S*	*K*
Item 1	3.96	2.18	−0.03	−1.35
Item 2	3.29	1.89	0.38	−0.96
Item 3	6.22	1.04	−1.62	2.90
Item 4	2.77	1.91	0.82	−0.46
Item 5	4.08	2.17	−0.15	−1.36
Item 6	4.47	1.90	−0.37	−0.99
Item 7	4.51	2.09	−0.37	−1.20
Item 8	5.78	1.38	−1.32	1.64
Item 9	4.05	2.07	−0.04	−1.29
Item 10	3.62	2.17	0.18	−1.42
Item 11	2.39	1.80	1.18	0.23
Item 12	5.60	1.50	−1.10	0.57
Item 13	5.00	1.79	−0.74	−0.37
Item 14	6.18	1.09	−1.61	2.89
Item 15	3.92	2.12	−0.08	−1.34
Item 16	5.46	1.65	−1.15	0.58
Item 17	4.16	1.92	−0.19	−1.07
Item 18	6.20	1.03	−1.27	1.00
Item 19	6.48	0.96	−2.41	6.67
Item 20	3.85	2.00	0.02	−1.20
Item 21	3.30	2.06	0.42	−1.08
Item 22	5.34	1.63	−0.92	0.11
Item 23	3.94	1.96	−0.06	−1.20
Item 24	3.38	2.01	0.38	−1.12
Item 25	5.79	1.36	−1.24	1.12
Item 26	5.34	1.66	−0.98	0.21
Item 27	5.97	1.27	−1.41	1.64
Item 28	4.40	2.11	−0.30	−1.24
Item 29	5.11	1.71	−0.71	−0.39
Item 30	5.33	1.64	−0.97	0.28
Multivariated	-	-	163.33	1137.70

*M* = mean; *SD* = standard deviation; *S* = skewness; *K* = kurtosis.

**Table 2:** Goodness of fit indices for the tested model of the VFI with CFA.

Models/Indices	$\chi^{2}/df$	CFI	TLI	RMSEA	RMSEA 90% CI	SRMR
1-Factor	6.002	0.895	0.887	0.103	0.100–0.107	0.118
2-Interrelated Factor	5.756	0.900	0.892	0.101	0.097–0.105	0.115
2-Independent Factor	10.351	0.803	0.788	0.142	0.138–0.145	0.157
4-Factors	3.143	0.959	0.954	0.068	0.062–0.073	0.085
5-Factors	2.937	0.959	0.954	0.064	0.059–0.070	0.082
6-Interrelated Factor	2.299	0.974	0.971	0.053	0.048–0.057	0.073
6-Interrelated Factor ^(a)^	2.568	0.968	0.965	0.058	0.054–0.062	0.077
6-Independent Factor	31.951	0.348	0.299	0.257	0.254–0.261	0.262
6-Independent Factor 1st-order and 2nd-Order	3.026	0.958	0.954	0.066	0.062–0.070	0.087

^(a)^ Six interrelated factor with item 29 reconfigured to the social factor; *χ*^2^/*df* = Chi-square/degree of freedom; CFI = comparative fit index; TLI = Tucker–Lewis fit index; RMSEA = root mean square error of approximation; CI = confidence interval; SRMR = standardized root mean square residual.

**Table 3:** Loadings of the VFI tested with CFA.

Factors	Items	Unstandardized	St. Error	*z*-Value	P(>|*z*|)	Standardized
F1	Item 3	1.00				0.41
	Item 8	1.87	0.14	13.88	0.00	0.57
	Item 16	2.44	0.17	14.58	0.00	0.63
	Item 19	1.07	0.08	13.00	0.00	0.47
	Item 22	2.65	0.18	14.75	0.00	0.68
F2	Item 12	1.00				0.65
	Item 14	0.77	0.04	21.42	0.00	0.68
	Item 18	0.63	0.03	20.26	0.00	0.59
	Item 25	0.96	0.05	21.44	0.00	0.69
	Item 30	1.39	0.06	23.10	0.00	0.83
F3	Item 2	1.00				0.55
	Item 4	1.32	0.06	21.67	0.00	0.71
	Item 6	1.22	0.06	20.80	0.00	0.66
	Item 17	1.48	0.07	22.14	0.00	0.80
	Item 23	1.71	0.08	22.78	0.00	0.90
F4	Item 1	1.00				0.76
	Item 10	1.09	0.04	27.39		0.83
	Item 15	1.08	0.04	27.25		0.84
	Item 21	1.03	0.04	27.30		0.82
	Item 28	1.03	0.04	27.08		0.80
F5	Item 7	1.00				0.79
	Item 9	0.97	0.04	26.71		0.77
	Item 11	0.56	0.03	21.72		0.51
	Item 20	0.91	0.03	26.45		0.75
	Item 24	1.00	0.04	27.49		0.82
F6	Item 5	1.00				0.67
	Item 13	0.79	0.03	25.98		0.64
	Item 26	0.79	0.03	26.61		0.70
	Item 27	0.43	0.02	21.91		0.50
	Item 29	0.79	0.03	26.33		0.68

ST. Error = standard-deviation error; *z*-value = Wald statistics; P(>|*z*|) = *p*-value.

**Table 4:** VFI Cronbach’s alphas, mean inter-item correlations, and corrected item-total correlation ranges.

VFI Sub-Scales	Alpha	CR	MIIC	CITCR
Values	0.69	0.70	0.34	0.43–0.53
Understanding	0.81	0.82	0.49	0.57–0.67
Social	0.85	0.84	0.53	0.61–0.76
Career	0.91	0.90	0.66	0.74–0.77
Protective	0.85	0.87	0.52	0.42–0.77
Enhancement	0.77	0.77	0.42	0.46–0.66

Alpha = Cronbach’s alpha; CR = composite reliability; MIIC = mean inter-item correlation; CITCR = corrected item-total correlation range.

**Table 5:** VFI factors correlation matrix and average variance extracted.

Factor	AVE	Correlation Matrix					
		F1	F2	F3	F4	F5	F6
F1	0.36	(0.60)					
F2	0.50	0.81	(0.70)				
F3	0.54	0.42	0.41	(0.74)			
F4	0.66	0.27	0.55	0.50	(0.81)		
F5	0.56	0.43	0.46	0.64	0.48	(0.75)	
F6	0.43	0.68	0.77	0.66	0.64	0.75	(0.67)

AVE = average variance extracted; diagonal elements in brackets = square root of AVE.

**Table 6:** VFI factors correlation, standard error, z value, 95% lower confidence interval, and 95% upper confidence interval.

Factors		*r*	SE	*z*	*p*-Value	LL	UL
1	2	0.808	0.041	19.819	0	0.728	0.888
1	3	0.419	0.027	15.713	0	0.367	0.471
1	4	0.268	0.022	12.088	0	0.224	0.311
1	5	0.434	0.027	16.180	0	0.382	0.487
1	6	0.678	0.037	18.260	0	0.605	0.751
2	3	0.406	0.021	19.111	0	0.364	0.447
2	4	0.551	0.021	26.715	0	0.511	0.592
2	5	0.457	0.022	21.110	0	0.414	0.499
2	6	0.767	0.032	23.781	0	0.704	0.830
3	4	0.497	0.018	27.201	0	0.462	0.533
3	5	0.636	0.022	29.410	0	0.594	0.679
3	6	0.664	0.026	25.650	0	0.613	0.715
4	5	0.484	0.018	26.478	0	0.448	0.519
4	6	0.640	0.023	27.621	0	0.594	0.685
5	6	0.746	0.027	27.578	0	0.693	0.799

LL = 95% lower limit; UL = 95% upper limit.

**Table 7:** Correlations between the VFI, organizational commitment, empathy, volunteer satisfaction, and age.

VFI/Other Subscales	OCQ Affective	OCQ Cognitive	OCQ Compromise	UWES	VSS	Age
Values	0.37 **	0.23 **	0.36 **	0.33 **	0.32 **	−0.04
Understanding	0.50 **	0.16 *	0.42 **	0.37 **	0.47 **	−0.18 **
Social	0.22 **	−0.01	0.18 *	0.20 **	0.29 **	−0.06
Career	0.23 **	−0.10	0.14	0.15 *	0.32 **	−0.50 **
Protective	0.22 **	−0.14	0.12	0.19 *	0.27 **	−0.09 *
Enhancement	0.52 **	0.03	0.41 **	0.28 **	0.45 **	−0.15 **

OCQ = Organizational Commitment Questionnaire; UWES = Utrecht Work Engagement Scale; VSS = Volunteer Satisfaction Survey; * *p* < 0.01; ** *p* < 0.05.

**Table 8:** Descriptive statistic and comparisons between genders and VFI sub-scales.

VFI	Total (*N* = 468)			Female (*n* = 357)		Male (*n* = 111)		*t*	*p*	*d*
	M	SD	Range	M	SD	M	SD			
Values	5.86	0.91	1.40–7.00	5.86	0.92	5.83	0.90	−0.36	0.716	−0.04
Understanding	5.82	1.02	2.00–7.00	5.86	1.03	5.70	0.98	–1.42	0.079	−0.15
Social	3.73	1.52	1.00–7.00	3.62	1.48	4.06	1.60	2.68	0.004	0.29
Career	3.84	1.81	1.00–7.00	3.88	1.82	3.71	1.80	−0.91	0.183	−0.10
Protective	3.64	1.57	1.00–7.00	3.57	1.57	3.83	1.57	1.52	0.065	0.17
Enhancement	5.10	1.27	1.00–7.00	5.04	1.26	5.28	1.27	1.74	0.042	0.19

*M* = mean; *SD* = standard deviation; *t* = statistic test; *p* = *p*-value; *d* = effect size.

## Data Availability

The data can be made available for consultation upon request from the corresponding author.
